# Predicting Outcomes in Men With Metastatic Nonseminomatous Germ Cell Tumors (NSGCT): Results From the IGCCCG Update Consortium

**DOI:** 10.1200/JCO.20.03296

**Published:** 2021-04-06

**Authors:** Silke Gillessen, Nicolas Sauvé, Laurence Collette, Gedske Daugaard, Ronald de Wit, Costantine Albany, Alexey Tryakin, Karim Fizazi, Olof Stahl, Jourik A. Gietema, Ugo De Giorgi, Fay H. Cafferty, Aaron R. Hansen, Torgrim Tandstad, Robert A. Huddart, Andrea Necchi, Christopher J. Sweeney, Xavier Garcia-Del-Muro, Daniel Y. C. Heng, Anja Lorch, Michal Chovanec, Eric Winquist, Peter Grimison, Darren R. Feldman, Angelika Terbuch, Marcus Hentrich, Carsten Bokemeyer, Helene Negaard, Christian Fankhauser, Jonathan Shamash, David J. Vaughn, Cora N. Sternberg, Axel Heidenreich, Jörg Beyer

**Affiliations:** ^1^ Oncology Institute of Southern Switzerland (IOSI), Bellinzona, Switzerland; ^2^ Universita della Svizzera Italiana, Lugano, Switzerland; ^3^ University of Manchester, Manchester, United Kingdom; ^4^ European Organisation for Research and Treatment of Cancer, Brussels, Belgium; ^5^ Rigshospitalet, Copenhagen University Hospital, Copenhagen, Denmark; ^6^ Erasmus MC Cancer Institute, Rotterdam, the Netherlands; ^7^ Indiana University Melvin and Bren Simon Cancer Center, Indianapolis, IN; ^8^ N.N. Blokhin Russian Cancer Research Center, Moscow, Russian Federation; ^9^ Research Institute of Oncology at Bashkir State Medical University, Ufa, Russian Federation; ^10^ Institut Gustave Roussy, University of Paris Saclay, Villejuif, France; ^11^ Department of Oncology, Skåne University Hospital, Lund, Sweden; ^12^ University Medical Center Groningen, Groningen, the Netherlands; ^13^ Istituto Scientifico Romagnolo per lo Studio e la Cura dei Tumori (IRST) IRCCS, Meldola, Italy and the Italian Germ Cell Cancer Group (IGG); ^14^ Medical Research Council Clinical Trials Unit, University College London (UCL), London, United Kingdom; ^15^ Division of Medical Oncology and Hematology, Princess Margaret Cancer Centre, University Health Network, Toronto, Ontario, Canada; ^16^ The Cancer Clinic, St Olavs University Hospital and Department of Clinical and Molecular Medicine, The Norwegian University of Science and Technology, Trondheim, Norway; ^17^ Institute of Cancer Research, Sutton, United Kingdom; ^18^ Fondazione IRCCS Istituto Nazionale dei Tumori, Milan, Italy. Current Affiliation: Vita-Salute San Raffaele University and IRCCS San Raffaele Hospital and Scientific Institute, Milan, Italy; ^19^ Department of Medical Oncology, Dana-Farber Cancer Institute, Boston, MA; ^20^ Catalan Institute of Oncology, IDIBELL Institute of Research, University of Barcelona, Barcelona, Spain; ^21^ Tom Baker Cancer Centre, University of Calgary, Calgary, Alberta, Canada; ^22^ Department of Medical Oncology and Hematology, University Hospital Zurich, Zurich, Switzerland; ^23^ Department of Urology, University Hospital Dusseldorf, Dusseldorf, Germany; ^24^ 2nd Department of Oncology, Faculty of Medicine, Comenius University and National Cancer Institute, Bratislava, Slovakia; ^25^ Division of Medical Oncology, Western University and London Health Sciences Centre, London, Ontario, Canada; ^26^ Australian and New Zealand Urogenital and Prostate Cancer Trials Group, Sydney, Australia; ^27^ Memorial Sloan Kettering Cancer Center, New York, NY; ^28^ Weill Medical College of Cornell University, New York, NY; ^29^ Division of Oncology, Department of Internal Medicine, Medical University of Graz, Graz, Austria; ^30^ Department of Hematology and Oncology, Red Cross Hospital, University of Munich, Munich, Germany; ^31^ Department of Oncology, Hematology and BMT with Section Pneumology, University Medical Center Hamburg-Eppendorf, Hamburg, Germany; ^32^ Department of Oncology, Oslo University Hospital, Oslo, Norway; ^33^ University of Zurich, Zurich, Switzerland; ^34^ St Bartholomew's Hospital, London, United Kingdom; ^35^ University of Pennsylvania, Philadelphia, PA; ^36^ Medical Oncology, San Camillo Forlanini Hospital, Rome, Italy. Current Affiliation: Englander Institute for Precision Medicine, Weill Cornell Medicine, New York-Presbyterian, NY; ^37^ Department of Urology, Uro-Oncology, Robot-Assisted and Specialized Urologic Surgery, University Hospital Cologne, Cologne, Germany; ^38^ University Department of Medical Oncology, Inselspital, University Hospital, University of Bern, Bern, Switzerland

## Abstract

The classification of the International Germ Cell Cancer Collaborative Group (IGCCCG) plays a pivotal role in the management of metastatic germ cell tumors but relies on data of patients treated between 1975 and 1990.

**MATERIALS AND METHODS:**

Data on 9,728 men with metastatic nonseminomatous germ cell tumors treated with cisplatin- and etoposide-based first-line chemotherapy between 1990 and 2013 were collected from 30 institutions or collaborative groups in Europe, North America, and Australia. Clinical trial and registry data were included. Primary end points were progression-free survival (PFS) and overall survival (OS). The survival estimates were updated for the current era. Additionally, a novel prognostic model for PFS was developed in 3,543 patients with complete information on potentially relevant variables. The results were validated in an independent data set.

**RESULTS:**

Compared with the original IGCCCG publication, 5-year PFS remained similar in patients with good prognosis with 89% (87%-91%) versus 90% (95% CI, 89 to 91), but the 5-year OS increased from 92% (90%-94%) to 96% (95%-96%). In patients with intermediate prognosis, PFS remained similar with 75% (71%-79%) versus 78% (76%-80%) and the OS increased from 80% (76%-84%) to 89% (88%-91%). In patients with poor prognosis, the PFS increased from 41% (95% CI, 35 to 47) to 54% (95% CI, 52 to 56) and the OS from 48% (95% CI, 42 to 54) to 67% (95% CI, 65 to 69). A more granular prognostic model was developed and independently validated. This model identified a new cutoff of lactate dehydrogenase at a 2.5 upper limit of normal and increasing age and presence of lung metastases as additional adverse prognostic factors. An online calculator is provided (https://www.eortc.org/IGCCCG-Update).

**CONCLUSION:**

The IGCCCG Update model improves individual prognostication in metastatic nonseminomatous germ cell tumors. Increasing age and lung metastases add granularity to the original IGCCCG classification as adverse prognostic factors.

## INTRODUCTION

About half of the patients with nonseminomatous germ cell tumors (GCT) (nonseminomatous germ cell tumors [NSGCT]) present with metastatic disease. Their cure rate is highly variable depending on histology, primary tumor location, tumor marker levels, and metastatic sites. In 1997, the International Germ Cell Cancer Collaborative Group (IGCCCG) published a classification, which became the accepted international standard and replaced all previous ones.^1^

In recent years, improved survival rates in metastatic NSGCT have been reported, possibly because of improved diagnostic tools, improved supportive care, introduction of the IGCCCG prognostic classification and tailored treatment according to this classification, better guideline adherence with standard use of cisplatin- and etoposide-based first-line treatments, more stringent use of postchemotherapy surgery, improved salvage treatments, and centralized management at dedicated expert centers or a combination of these factors.^2–7^

According to the original IGCCCG classification, metastatic NSGCT are split into good, intermediate, and poor prognostic categories based on levels of alpha-fetoprotein (AFP), human chorionic gonadotropin (hCG), and lactate dehydrogenase (LDH) as well as the presence of nonpulmonary visceral metastases (NPVM). In addition, all primary mediastinal NSGCT are classified as poor, irrespective of other factors.^1^ However, patients included in the original IGCCCG analysis were treated between 1975 and 1990, and not all had received cisplatin or etoposide, which would be the treatment backbone for metastatic NSGCT today.^3,7^

The IGCCCG Update Consortium collected data on metastatic NSGCT with two major goals: first, to validate the original IGCCCG criteria and update survival probabilities in a modern cohort and second, to explore additional prognostic factors that may add granularity to the original IGCCCG prognostic groups and explain some of the heterogeneities found within the groups of the original IGCCCG classification.^8^

## MATERIALS AND METHODS

### The IGCCCG Update Consortium

The IGCCCG Update Consortium consisted of 30 institutions or collaborative groups in Europe, North America, and Australia. Potential contributors were identified through contact between peers, supplemented by a PUBMED search. The principal investigators of individual trials were invited to participate in the initiative based on a written data sharing agreement.

In addition, the coordinators of national cooperative groups in GCT or principal physicians at large cancer centers were contacted with respect to the availability of national or local cancer registries in electronic format. Investigators were asked to contribute consecutive patients. The Protocol (online only) and the list of collected data items are available in the Data Supplement (online only).

### Data Collection

The purpose of the collaboration was to establish a common electronic database with data of patients with metastatic GCT treated between 1990 and 2013: the IGCCCG Update Data Warehouse. To ensure appropriate representation of patients and because trials often limit eligibility to specific IGCCCG prognostic groups, structured data from national registries, databases, or large cohorts of single center data on consecutively treated patients who fulfilled the Protocol data and eligibility requirements were collected in addition to data from clinical trials.

After signing of the data sharing agreements, patient‐level data were aggregated, normalized, and harmonized. Data were processed centrally and stored in a secure format at the headquarters of the European Organisation of Research and Treatment of Cancer in Brussels, Belgium.

### Patients and Data

Thirty participating members of the IGCCCG consortium provided anonymized data on consecutive adult male patients with metastatic NSGCT or primary retroperitoneal or mediastinal NSGCT also when not metastatic.

All patients had to receive cisplatin- and etoposide-based conventional-dose first-line treatment or upfront high-dose chemotherapy requiring stem-cell support for NSGCT. Patients with prior chemotherapy for metastatic disease, those included in the original IGCCCG analysis, and patients with primary GCT of the brain were ineligible. Patients treated with conventional chemotherapy had to receive minimum three cycles; patients with less than three cycles were allowed, provided that there was enough evidence that at least three cycles were intended. The treatment intended to be given to the patients was recorded where available, and treatments actually given otherwise.

Data items included the original IGCCCG group, age, date of metastatic diagnosis, and primary site; levels of serum AFP, hCG, and LDH at diagnosis and before chemotherapy, and the presence and location of metastases. The type and number of chemotherapy cycles were obtained, and progression status, vital status, cause of death, and disease status at last follow-up were recorded.

### Trials and Cohorts

We asked for electronic databases of studies and cohorts comprising a minimum of 100 eligible patients for inclusion in the warehouse. Only databases of first-line chemotherapy as described in the patient eligibility criteria were included. Retrospective data of first-line treatments of patients who were primarily referred for relapse were not included, because it would have artificially inflated the progression probabilities in the data warehouse.

### End Points

Primary end points were progression-free survival (PFS) and overall survival (OS). OS was defined as the time from start of chemotherapy to death of any cause. PFS was defined from start of chemotherapy to progression, defined by radiological progression, unequivocal tumor marker increase, or death, whichever came first. PFS was used for the prognostic model training.

### Statistical Methods

All patients with available PFS and/or OS information were used to update the survival probabilities. Kaplan-Meier estimates were used to update survival estimates according to the original IGCCCG. 95% CI are provided via log-log transform.^9,10^

To build a new prognostic model allowing for individual prediction, an analysis set consisting of eligible patients with all considered explanatory variables was created. These variables were the prechemotherapy AFP and hCG as continuous variables and LDH levels times upper limit of normal (ULN), site of primary tumor, age (in years) as a continuous variable, presence of NPVM and presence of lung metastases. Patients with unspecified other primary tumor site were excluded. The increase in risk because of AFP and hCG elevations was modeled only for unequivocal marker elevation, defined as AFP > 30 ng/mL and hCG > 5U/L, respectively. Values below those thresholds were considered equivocal and were grouped with values < ULN. Studies forming the analysis set were split into six major clusters based on geographical considerations to account for some of the heterogeneities between patient populations. Details on these clusters are given in the Data Supplement.

Two thirds of studies forming each cluster were included in the training set, whereas the remaining studies formed the independent validation set. Because of the lower number of events for OS, the prognostic IGCCCG Update model was developed for PFS, based on all patients in the training set with complete PFS information. Both end points were administratively censored at 3 years to harmonize duration of follow-up across data sources since most events had occurred by this time (Appendix, online only).

All candidate variables were used for model building. Continuous variables were assessed for linearity based on graphical checks and transformed or categorized if warranted. The experts from the steering committee identified a priori the most likely clinically relevant interactions to be considered as the interactions between the presence of NPVM and the level of the prechemotherapy markers (AFP, hCG, and LDH). These interactions were globally tested at the 5% significance level.

The final IGCCCG Update model is defined in two parts: (1) The prognostic score, which models the prognostic impact of each covariate on PFS. The prognostic score of the final IGCCCG Update model was obtained by incorporating all candidate variables in a Cox proportional hazards model, stratified on the six clusters previously specified. (2) The baseline hazard, which models the risk of progression as a function of time for patients who had baseline levels of all prognostic factors entered in the model. The baseline hazard for the final IGCCCG Update model was taken as the average of the six cluster-specific baseline hazard using a Royston-Parmar parametric model, with the score previously obtained as sole covariate.^11^

The performance of the final IGCCCG Update model was investigated in the training and validation set. Overall performance over time was assessed using an integrated Brier score (IBS), which integrates the apparent estimate of the prediction error over 3 years.^12^ Prediction accuracy was assessed via calibration plots, whereas the discriminative ability of the prognostic score was evaluated using time-dependent area under the curve (AUC).^13^

The final IGCCCG Update model was graphically represented via nomograms.

All analyses were performed using SAS version 9.4 (Cary, NC), R software (version 3.6.0), and Stata version 13 (StataCorp, Texas).

## RESULTS

### Patient Characteristics

**FIG 1.**
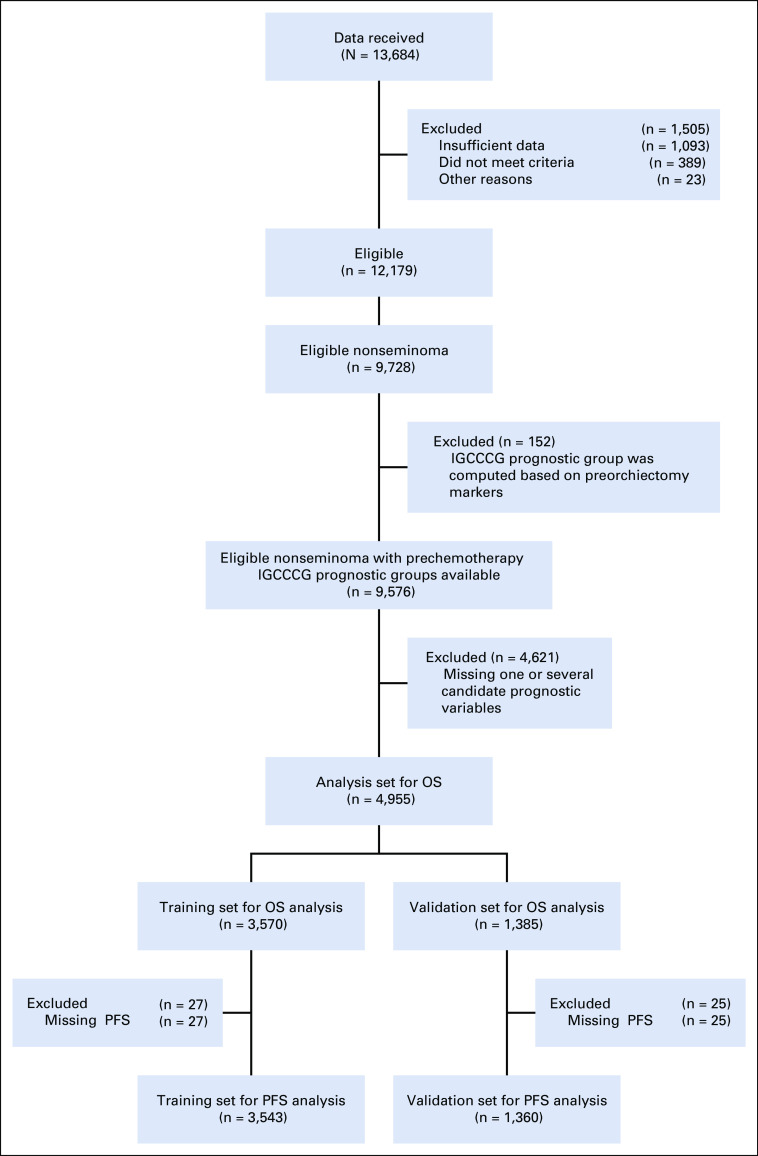
CONSORT diagram. IGCCCG, International Germ Cell Cancer Collaborative Group; OS, overall survival; PFS, progression-free survival.

In total, data on 13,684 patients with GCT were received, of whom 12,179 (89%) were eligible. Of these patients, 9,728 had been diagnosed as NSGCT based on histology and/or unequivocal AFP elevations. Reasons for ineligibility are listed in the CONSORT diagram (Fig 1). The original IGCCCG groups could be calculated in 9,576 of 9,728 (98%) patients, whose data were used to update OS probabilities. Among them, 7,313 patients were initially recorded in local or national cohorts, whereas 2,263 came from clinical trial databases. Because of inconsistent or missing data, 9,420 (99%) patients were used to update PFS probabilities.

Disease progression occurred in 2,190 patients, and 1,352 patients died. The PFS median follow-up was 6.4 years (7.4 years for cohorts and 3.8 years for trials), and 81% had been followed for at least 3 years from start of chemotherapy (87% for cohorts and 64% for trials).

The analysis set consisted of 4,955 patients with NSGCT, in whom potentially relevant covariates were available. The analysis set was split between a training set of 3,570 patients (72%, 3,543 with PFS information) and a validation set of 1,385 patients (28%, 1,360 with PFS information).

Table 1 shows the baseline characteristics of the 4,955 patients in the analysis set, divided between training and validation sets. Slightly more patients in the validation set had NPVM (poor prognosis IGCCCG) compared with the training set. Additionally, the patients in the validation set were treated more recently compared with patients in the training set (83.6% of patients in the validation set were treated after 2,000 *v* 60.5% in the training set). Finally, 97.6% of all trial patients were allocated to the training set.

**TABLE 1.**
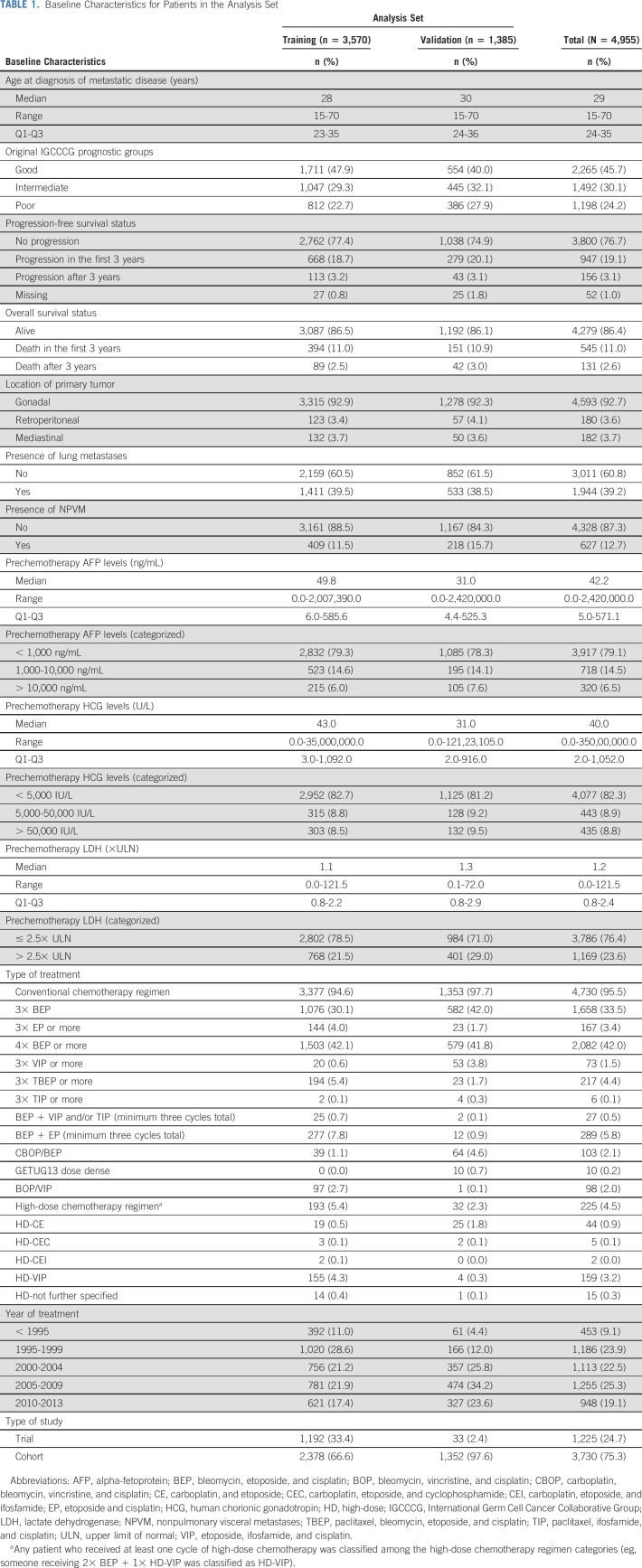
Baseline Characteristics for Patients in the Analysis Set

A comparison between patients included or not included in the prognostic model analysis set showed that the most common reason for exclusion was missing information about LDH (75.8%) (Data Supplement) and that there were no differences in PFS or OS between patients included in or excluded from the analysis set (Data Supplement).

### Updated Outcomes by Original IGCCCG

**FIG 2.**
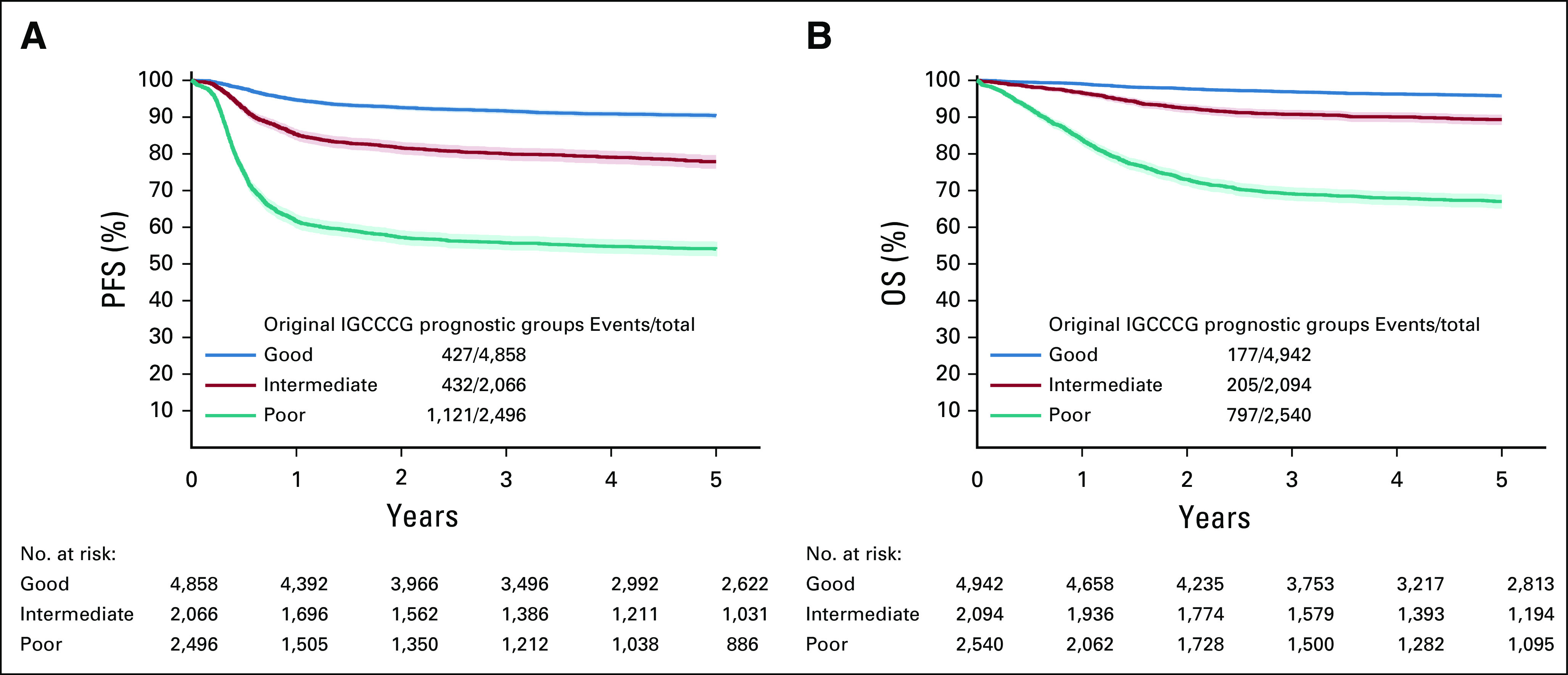
Survival probabilities and 95% CI according to original IGCCCG prognostic groups for (A) PFS and (B) OS. IGCCCG, International Germ Cell Cancer Collaborative Group; OS, overall survival; PFS, progression-free survival.

Table 2 shows the updated 5-year OS and PFS probabilities in the present series as compared with those in the original IGCCCG publication.^1^ Corresponding Kaplan-Meier curves of OS and PFS are presented in Figures 2A and 2B.

**TABLE 2.**
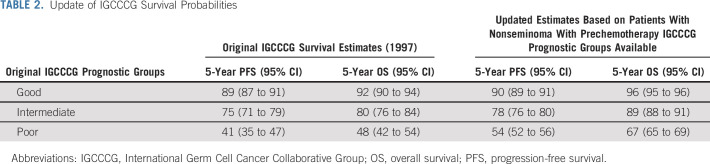
Update of IGCCCG Survival Probabilities

The 5-year OS significantly improved for all IGCCCG groups, as shown by the nonoverlapping confidence intervals. In contrast, 5-year PFS significantly improved only for poor IGCCCG patients.

### New Prognostic IGCCCG Update Model for Individual Prediction of PFS

The training set for the prognostic model included 3,543 patients with complete information on potentially important prognostic variables. Graphical assessment of linearity (Data Supplement) showed that the effect of age on progression was linear, whereas the effect of AFP and hCG was log-linear (log-2 transformation).

The ULN of LDH proved to have a relationship with PFS akin to a three-step function with cut points located close to 1× ULN and 2.5× ULN (Data Supplement). As such, it was categorized, using the single threshold of 2.5× ULN, based on clinical relevance.

All candidate interactions were included in the model (global test *P* < .0001).

The final IGCCCG Update model is presented in Table 3. Consistent with the original classification, the presence of NPVM (hazard ratio [HR] = 6.61 [95% CI, 4.62 to 9.46]) and a mediastinal primary tumor (HR = 2.68 [95% CI, 2.04 to 3.53]) were the most important prognostic factors. The IGCCCG Update model also highlights two new adverse prognostic variables. Every decade-of-life increase translates into a 25% increase in the risk of progression. The presence of lung metastases translates into a 62% increase in the risk of progression compared with patients without lung metastases. The final IGCCCG Update model was robust across year of treatment, geographical region, and trial versus nontrial patients (data not shown).

**TABLE 3.**
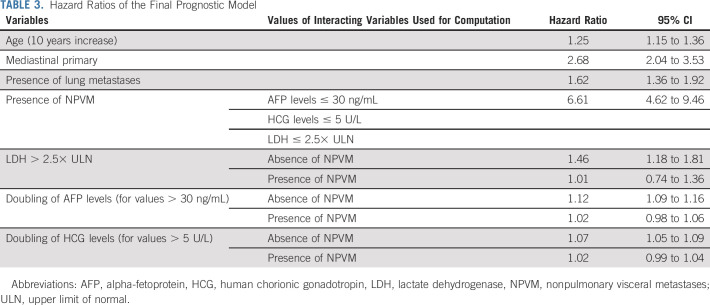
Hazard Ratios of the Final Prognostic Model

### Nomograms for Calculating Prognosis

**FIG 3.**
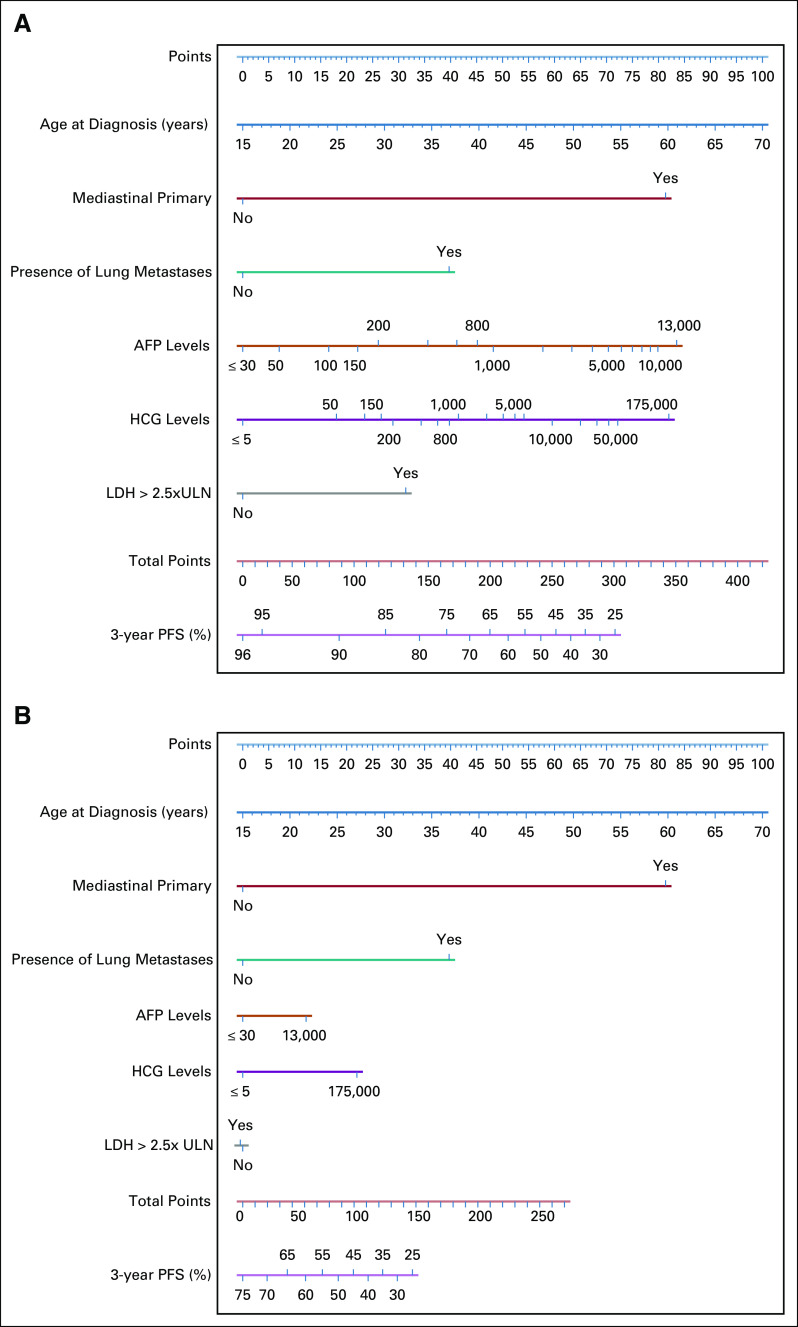
Nomograms of the final prognostic model for patients (A) without NPVM and (B) with NPVM. The extreme values of AFP and HCG markers on the nomograms were truncated to the rounded 95% percentile of the training set (13,000 ng/mL for AFP, 175,000 U/L for HCG). Predicted 3-year PFS below 25% are not shown, as it corresponds to a combination of baseline prognostic factors rarely seen in the training set. AFP, alpha-fetoprotein, HCG, human chorionic gonadotropin, LDH, lactate dehydrogenase, NPVM, nonpulmonary visceral metastases; PFS, progression-free survival; ULN, upper limit of normal.

For improved readability, the graphical representation of the final IGCCCG Update model is presented in two separate nomograms: one for patients with NPVM and the other for patients without NPVM. These are shown in Figure 3 and made available as a web application (https://www.eortc.org/IGCCCG-Update).^14^

### Performance of the IGCCCG Update Prognostic Model

The IBS is a measure of prediction error ranging from 0 (perfect accuracy) to 1 (totally inaccurate). The IBS in the training set was 0.10.

The time-dependent 3-year AUC was 0.76 (95% CI, 0.73 to 0.78), showing the ability of the prognostic score to accurately rank patients' risk of progression based on their baseline prognostic factors (Data Supplement). The final IGCCCG Update model also demonstrated excellent calibration to predict 3-year PFS in the training set (Data Supplement).

### Independent Validation

In the validation set, the IGCCCG Update model had an IBS of 0.11, a 3-year AUC of 0.74 (95% CI, 0.70 to 0.77), and equally good calibration (Data Supplement).

## DISCUSSION

The international IGCCCG Update Consortium database is the largest source of information on metastatic GCT worldwide. In this report, we analyzed the data of 9,728 patients with metastatic NSGCT treated between 1990 and 2013. The majority of patients in the database were treated according to international guidelines with three cycles of bleomycin, etoposide, and cisplatin or four cycles of EP in patients with good prognosis and four cycles of bleomycin, etoposide, and cisplatin or equivalent conventional-dose regimens in patients with intermediate and poor prognosis.^15,16^ Only a minority of patients received dose-intensified regimen such as dose-dense or high-dose chemotherapy.^17–20^

Compared with the results of the original IGCCCG cohort, in this modern series, patients with NSGCT from all prognostic groups experienced substantially improved OS. The differences were most striking in patients with poor prognosis GCT, in whom OS and PFS improved by 19% and 13%, respectively. By contrast, PFS among patients with good and intermediate prognosis NSGCT was only slightly better than that in the original IGCCCG report. This suggests that improved first-line treatment (chemotherapy and surgery) might have had the greatest impact on patients with poor prognosis NSGCT, whereas all prognostic groups benefitted from more effective salvage strategies.

This large multicenter database capturing modern type treatments over a period of more than 20 years confirms previous smaller series that also reported better outcomes in more recently treated patients with NSGCT.^2,5,19,21^ These improvements might have resulted from stage migration because of earlier diagnosis and better diagnostic tools, improved supportive care, superiority of cisplatin- and etoposide-based first-line treatment over other combinations, use of upfront dose-intensified regimens, more stringent use and higher quality of postchemotherapy surgery, better salvage strategies in nonresponding or relapsing patients, more stringent guideline adherence, centralization of care at experienced expert centers, or a combination of these factors.^2–7,16^ Additionally, the availability of the original IGCCCG classification itself helped to resolve the confusion from heterogenous previous classifications and to guide appropriate treatment duration.^22^ Given 5-year PFS and OS survival probabilities of 78% (95% CI, 77 to 79) and 87% (95% CI, 86 to 87) across all prognostic groups, metastatic NSGCT is, together with seminoma, the most curable metastatic solid cancer in males.

An important finding of the present analysis is that the original IGCCCG classification as published in 1997 still distinguishes three prognostic groups among patients with metastatic NSGCT with significantly different PFS and OS probabilities. However, our model highlights the considerable heterogeneity in prognosis within the original IGCCCG groups, as shown in the Data Supplement. Moreover, we identified increasing age and the presence of lung metastases as additional adverse prognostic factors that could explain some of these heterogeneities. Additionally, the strong negative prognostic impact of marker elevation seen in patients without NPVM becomes much less relevant when NPVM is present.

The original IGCCCG classification used three categories of LDH elevation. Elevations above 10× ULN were infrequent in our series. We found 2.5× ULN to be the most clinically relevant cutoff value having a high specificity.

Our findings confirm reports of smaller studies that also suggested age and the presence of lung metastases as adverse prognostic factors.^5,23,24^ In particular, the new IGCCCG Update model shows that the negative prognostic impact of age can, in some cases, be more important than any other prognostic factor, with the exception of the presence of NPVM and primary mediastinal GCT. The reasons for the adverse prognosis with advanced age are unknown and seem to be not related to an increased treatment-related mortality. In our series, there was no evidence that patients' age influenced the choice of treatment. Dose reductions or treatment delays may be responsible for the adverse effect of age, but these particular data were not collected. Focusing on the treatment of older patients with germ cell tumor should therefore become a priority for further prospective studies.

Inadequate tumor marker decline is another validated adverse prognostic factor that identified patients with poor prognosis NSGCT who benefitted from treatment intensification.^19^ However, postchemotherapy marker decline was not captured in sufficient numbers in the IGCCCG Update database to be incorporated in the multivariate IGCCCG Update model.

As available trial data used the original IGCCCG classification for treatment stratification, we suggest that this classification remains the reference standard for treatment decisions in daily practice. However, a nomogram adding the two new variables, presence of lung metastases and age, as well as a new LDH cutoff of 2.5× ULN instead of 1.5× ULN allows for an improved and more granular individual prognostic assessment in patients with first-line metastatic NSGCT. Although this tool appears more complex than the original IGCCCG classification, it can be easily accessed via a web-based application (https://www.eortc.org/IGCCCG-Update).^14^ In future trials, patients with a particularly favorable prognosis in the nomogram may be subjected to de-escalation strategies to further reduce treatment burden in patients likely to be cured. In contrast, trials evaluating dose-escalation strategies should be pursued in patients with the worst prognosis according to the new IGCCCG Update model.

In conclusion, OS of patients with first-line metastatic NSGCT has improved over the last 20 years. However, despite these improvements, more than 30% of patients with poor prognostic features may still die of their disease. The original IGCCCG classification retains its relevance as a reference for treatment decisions in daily practice. The new IGCCCG Update model includes age and lung metastases as additional adverse prognostic factors and uses a single cutoff of LDH at 2.5× ULN. A web-based calculator (https://www.eortc.org/IGCCCG-Update) based on the results of the IGCCCG Update analysis allows improved and more granular individual prognostic assessment^14^ and can help to shape strategies for future trials.

## APPENDIX

We thank the following centers, research groups, and cancer registries who contributed data to the International Germ Cell Cancer Collaborative Group Update Consortium data warehouse:

### Australia

Australian and New Zealand Urogenital and Prostate Cancer Trials Group, Sydney and investigators of the P3BEP study (ACTRN12613000496718) and New Zealand Urogenital and Prostate Good prognosis GCT study (ACTRN12605000142639) trials;

### Canada

Princess Margaret Cancer Centre, University Health Network, Toronto, Ontario; Indiana Tom Baker Cancer Centre, University of Calgary, Calgary, Alberta; Western University and London Health Sciences Centre, London, Ontario.

### Europe

Belgium The European Organisation for Research and Treatment of Cancer (EORTC) Genito-Urinary Cancer Group and investigators of the EORTC 30895, EORTC 30941, EORTC 30974, and EORTC 30983 trials; The EORTC Headquarters, Brussels.

#### Austria.

Medical University of Graz.

#### Denmark.

Righshopitalet Copenhagen and contributors to the Danish Germ Cell Cancer Registry.

#### France.

Institut Gustave Roussy, University of Paris Saclay, Villejuif; Groupe d’Etude des Tumeurs Uro-Génitale (GETUG) and investigators of the GETUG-13, S99 and T93 trials.

#### Germany.

Universitätsklinikum Düsseldorf, Düsseldorf; Universitätsklinikum Köln/Köln University Hospital, Köln; The Red Cross, University of Munich, Munich; University Medical Center Hamburg-Eppendorf, Hamburg; The German Testicular Cancer Study Group and investigators of the HDVIP protocol (DOI: 10.1200/JCO.2003.09035).

#### Italy.

San Camillo Forlanini Hospital, Rome; Fondazione IRCCS Istituto Nazionale dei Tumori, Milano; The Italian Germ Cell Cancer Group (IGG).

#### Norway and Sweden.

The Norwegian University of Science and Technology, Trondheim, Norway; Oslo University Hospital, Oslo, Norway; Skåne University Hospital, Lund, Sweden; The Swedish and Norwegian Testicular Cancer Group (SWENOTECA).

#### Slovakia.

National Cancer Institute, Bratislava.

#### Spain.

Hospital Universitario Morales Meseguer-IMIB, Murcia; Universidad Católica San Antonio de Murcia (UCAM), Murcia; Institut Catala d'Oncologia, Barcelona; The Spanish Germ Cell Cancer Group and contributors of the SGCCCG registry and of the “chemotherapy versus radiotherapy study” (DOI: 10.1200/JCO.2007.15.9103)

#### Switzerland.

University of Zürich, Zürich.

#### The Netherlands.

University Medical Center Groningen, Groningen.

### The United Kingdom

The Institute of Cancer Research, Sutton; The Medical Research Council Testicular Cancer Study Group and investigators of the MRC TE13 and TE20 studies; St Bartholomew’s Hospital, London.

### Russia

N.N. Blokhin Russian Cancer Research Center, Moscow, Russian Federation.

### The United States of America

Indiana University, Melvin and Bren Simon Cancer Center, Indianapolis, Indiana; Dana-Farber Cancer Institute—Harvard Medical School, Boston, Massaschussets; University of Pennsylvania, Perelman Center for Advanced Medicine, Philadelphia, Pennsylvania; Memorial Sloan Kettering Cancer Center, New-York, New-York, USA and investigators of the MSKCC0747 and MSKCC94076 studies.
